# The Human Epidermal Growth Factor Receptor 2 Screening Tests for Breast Cancer Suggested by the New Updated Recommendation of the American Society of Clinical Oncology/College of American Pathologists Will Involve a Rise of the In-Situ Hybridization Tests for the European Laboratories of Pathology

**DOI:** 10.1155/2014/793695

**Published:** 2014-04-22

**Authors:** Christian Garbar, Aude-Marie Savoye, Corinne Mascaux, Eva Brabencova, Hervé Curé

**Affiliations:** Biopathology and Oncology Departments, Institut Jean Godinot, Unicancer, 1 rue du Général Koenig CS80014, 51726 Reims Cedex, France

## Abstract

*Aims*. The differences between the 2007 and the 2013 ASCO/CAP HER2 guidelines have been compared. We also discussed the potential consequences in our pathological practice. *Material and Methodology*. 189 HER2 fluorescence in situ hybridisation (FISH) tests were performed from 1016 preliminary HER2 immunohistochemical tests (IHC). All cases were reviewed and reclassed following the 2007 and 2013 ASCO/CAP recommendations. *Results*. The 2013 version decreased false-negative IHC (3/118 versus 1/54, *P* = ns) and created more 2+ IHC (40/186 versus 89/186, *P* = 0.001) or more 3+ IHC (9/186 versus 39/186, *P* = 0.001). One false-positive IHC was described for the 2013 version (0/9 versus 1/39, *P* = ns). Equivocal FISH was reduced (8/186 versus 2/186, *P* = ns). An estimation based on our data for 1000 patients showed a rise of our FISH tests for the control of 2+ IHC (180 tests for the 2007 version versus 274 tests for the 2013 version or FISH work overflow is +52%) and for the control of 2+/3+ IHC (300 for the 2007 version versus 475 for the 2013 version or FISH work overflow is +58%). *Conclusions*. The new 2013 ASCO/CAP guidelines have detected more HER2 positive cases but have increased the number of FISH tests.

## 1. Introduction

In Europe, the breast cancer incidence is 464.000 cases representing the most common cause in women [1]. Most of them are treated following the oestrogens receptors (ER), progesterone receptors (PR), and epidermal growth factor receptor type 2 (HER2) status [2].

The human epidermal growth factor receptor 2 (HER2) was first discovered in 1984. The HER2 gene encodes a transmembrane tyrosine kinase receptor protein involved in proliferation, tumoral invasion, angiogenesis, apoptosis, and metastasis. For equivalent clinical and pathological stage, patients benefiting of anti-HER2 therapy showed a better prognosis than HER2-negative patients [3].

In the literature, the HER2 gene is classically amplified in 9% to 74% of human breast cancers (mean 22%) [4]. In 1998, the trastuzumab treatment of positive HER2 invasive breast cancer associated with its companion immunohistochemistry test (IHC) was approved by the Food and Drug Administration.

Consequently, the HER2 status is actually based on IHC triage tests and chromogenic or fluorescence in situ hybridization (FISH) controlling the equivocal IHC results. The choice of IHC is based on its low cost, easy preservation of staining slides, and use of a familiar routine histology. Its disadvantages are the sensible preanalytic issues such as duration and type of fixation, intensity of antigen retrieval, type of antibody, lack of positive internal control signal, and difficulties of reproducible scoring. The FISH advantages are the presence of internal control and the more objective scoring system. The disadvantages are the higher cost and the time-consuming to perform the technique. The potential cause of false-positive FISH is to confuse in situ carcinoma area with invasive component. False-negative FISH results are rare and are often the consequence of the tumoral heterogeneity [4].

To improve the efficiency of the HER2 screening, the American Society of Clinical Oncology (ASCO) and the College of American Pathologists (CAP) edited, in 2007, recommendations to the HER2 pathological diagnosis, according to an equilibrated economic/benefits strategy of the anti-HER2 treatment [5].

One of disadvantages of this 2007 version was the true possibility to make false-negative IHC tests and nonnegligible number of equivocal FISH.

To answer to this reality, an important update of the ASCO/CAP HER2 recommendations has been recently published to improve the accuracy of the HER2 test [6].

The aim of this retrospective work is to compare the main differences between the 2007 and the 2013 ASCO/CAP HER2 guidelines and the potential consequences in our routine practice.

## 2. Materials and Methodology

### 2.1. Patient Population

Between January 2012 and June 2013, 217 HER2 fluorescence in situ hybridisation (FISH) tests were performed in our institution from 217 women (age: 62.5 ± 13.9 years) presenting with breast cancer. All cases were selected by preliminary HER2 immunohistochemical tests presenting from 1016 cases. FISH was indicated for equivocal results (2+: 175/217 (80.6%)) or for quality control (0 or 1+: 10/217 (4.6%) and 3+: 32/217 (14.7%)). For this study and according to the literature recommendations, 28 FISH tests were excluded because the fixation was nonoptimal (*n* = 14) and crush artefacts (*n* = 6), the tumoral tissue was too small to be correctly interpreted (*n* = 4), and the diagnosis was not appropriated such as in situ carcinoma with microinvasion (*n* = 4) [5, 6]. Finally, 189 cases were available for this study: 104 surgical samples and 85 core needle aspiration biopsies (CNB). The main characteristic of patients was the following: pT1b: 10/80 (12.5%), pT1c: 35/80 (43.7%), pT2-3: 35/80 (43.7%), pN0: 36/72 (50.0%), pN1-2: 36/72 (50.0%), SBR1: 17/171 (9.9%), SBR2: 87/171 (50.8%), and SBR3: 67/171 (39.1%). The prevalence of positive HER2 in our population of 1016 women calculated with the 2007 recommendations was of 15.1%.

### 2.2. Histological Procedures

All surgical specimens were initially fixed in 4% buffered formaldehyde solution between 6 and 48 hours and then imbedded in paraffin and cut to 4 *μ*m. The slides were stained with a classical haematoxylin-eosin stain to perform the initial diagnosis.

Immunohistological staining was performed with the Dako Autostainer Link 48 immunostaining system (Dako Glostrub, Denmark) using HER2 primary antibodies (clone A0485, Dako) and according to the manufacturer's instructions [7, 8].

HER2 IHC of all cases was confirmed by a rapid FISH technique using HER2/C17 probes (HER2 IQFISH pharm DX, Dako), according to the manufacturer's instructions. Briefly, specimen was denaturised at 66°C for 10 minutes. The hybridisation was performed for 90 minutes at 45°C simultaneously for HER2/Texas Red labelled DNA probe and CEN-17/FITC labelled DNA probe using a hybridizer device (Dako). We used a fluorescence microscope with appropriate filters (NIKON, Japan).

### 2.3. Quantification

All HER2 IHC were blindly and independently reviewed by CG and CM according to both the 2007 and the 2013 guidelines of the College of American Pathologists [5, 6]. Discordant results were secondarily discussed and the consensual diagnosis has been retained for the final result.

**Figure 1:**
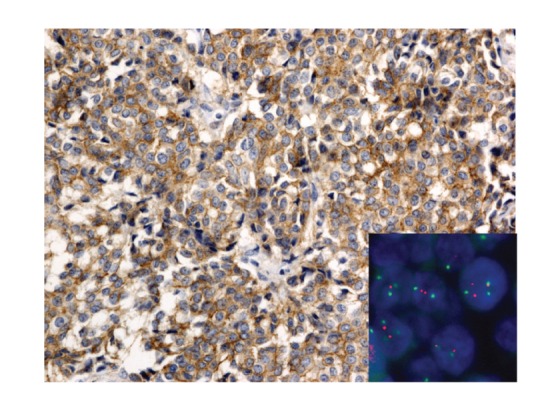
An 89-year-old woman with ductal breast carcinoma. IHC classified 1+ with the 2007 ASCO/CAP recommendation and 2+ with the 2013 ASCO/CAP version. Ratio between HER2 (red) and CEN-17 (green) is 2. FISH is equivocal for the 2007 system and negative for the 2013 because the mean of HER2 copy is inferior to 4. HER2 IHC (Clone A0485, Dako, 200x magnification) and HER2 FISH (HER2 IQFISH pharm DX, Dako, 1000x magnification).

**Figure 2:**
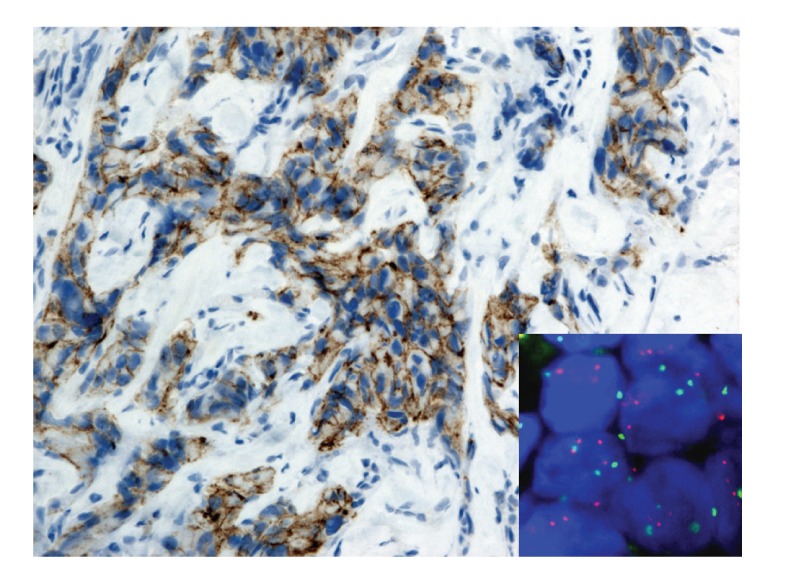
A 68-year-old woman with ductal breast carcinoma. IHC classified 1+ with the 2007 ASCO/CAP recommendation and 2+ with the 2013 ASCO/CAP version. Ratio between HER2 (red) and CEN-17 (green) is 1.9. FISH is equivocal for the 2007 system and equivocal for the 2013 because the mean of HER2 copy is 4.5. HER2 IHC (Clone A0485, Dako, 200x magnification) and HER2 FISH (HER2 IQFISH pharm DX, Dako, 1000x magnification).

**Figure 3:**
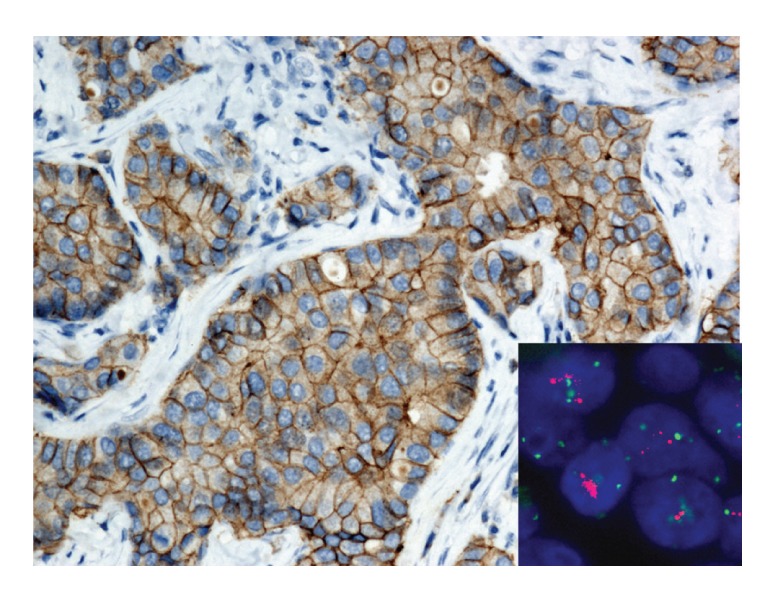
A 66-year-old woman with ductal breast carcinoma. IHC classified 2+ with the 2007 ASCO/CAP recommendation and 3+ with the 2013 ASCO/CAP version. Ratio between HER2 (red) and CEN-17 (green) is 1.9. FISH is equivocal for the 2007 system and positive for the 2013 because the mean of HER2 copy is 6.2. HER2 IHC (Clone A0485, Dako, 200x magnification) and HER2 FISH (HER2 IQFISH pharm DX, Dako, 1000x magnification).

**Figure 4:**
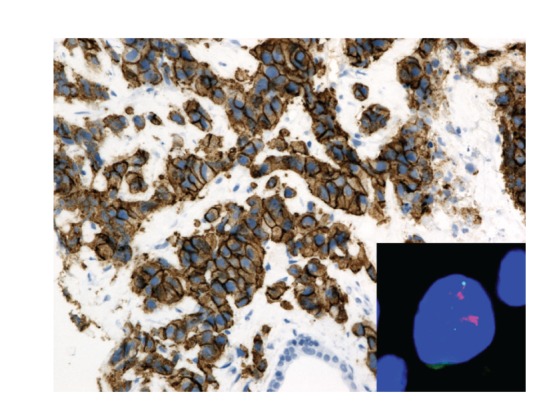
A 91-year-old woman with ductal breast carcinoma. IHC classified 2+ with the 2007 ASCO/CAP recommendation and 3+ with the 2013 ASCO/CAP version. Ratio between HER2 (red) and CEN-17 (green) is 5.3. FISH is positive for the 2007 system and positive for the 2013. HER2 IHC (Clone A0485, Dako, 200x magnification) and HER2 FISH (HER2 IQFISH pharm DX, Dako, 1000x magnification).

Briefly,* for 2007 version*, a positive HER2 test was defined as follows (Figures 1, 2, 3, and 4).

1. Positive IHC (3+) stains or more than 30% of tumor cells present intense and uniform circumferential membrane staining with a homogeneous and continuous cell positivity giving a “chicken wire pattern.” Incomplete or pale membrane staining is ignored.
2. Or a positive FISH presents a ratio between HER2 gene and chromosome 17 signals superior to 2.2.

Equivocal test is characterized as

1. IHC presenting a weak to moderate complete membrane in superior to 10% tumor cells (2+);
2. FISH ratio between 1.8 and 2.2.

A negative result is an IHC staining of 0/1+ and a FISH ratio inferior to 1.8.

*For 2013 version*, a positive HER2 test was defined as follows (Figures 1, 2, 3, and 4).

1. Positive IHC within more than 10% of tumor cells presents intense and uniform complete circumferential membrane staining with a homogeneous and continuous cell positivity giving a “chicken wire pattern” (3+). Incomplete or pale membrane staining is ignored.
2. Or a positive FISH presents a ratio between HER2 gene and chromosome 17 signals superior or equal to 2.0
3. Or a positive FISH presents an average of HER2 copy superior or equal to 6.0.

Equivocal test is characterized as

1. positive IHC within less than or equal to 10% of tumor cells presenting intense circumferential membrane staining (2+);
2. positive IHC within more than 10% of tumor cells presenting weak/moderate incomplete membrane staining (2+);
3. FISH ratio inferior to 2.0 with an average of HER2 copy superior or equal to 4.0 and inferior to 6.0 signals.

A negative result is an IHC staining of 0/1+ and a FISH ratio <2.0 and an average HER2 copy inferior to 4.0.

### 2.4. Statistics

ANOVAs tests were performed for parametric results and Fisher's exact test for nonparametric data. Kappa sensitivity and specificity statistical tests were evaluated by the Analyse-it 2.30 (Leeds, UK) and Excel 2003 (Microsoft Corp., Redmond, Washington, USA) programs. A* P*  value <0.05 was considered significant.

## 3. Results

In the 2007 guidelines (Table 1), 137 cases were negative in which there were 3 false-negative IHC. Equivocal IHC (2+) was HER2 gene amplified on FISH in 52.5% (21/40). No false-positive IHC has been observed for 3+. These results illustrated the weakness of the 2007 guidelines creating some false-negative results that have been discussed in the 2013 version [6]. 8 FISH tests were considered as equivocal, mainly for 1+ and 2+ but not for 3+.

The 2013 version (Table 1) created more positive results for the HER2 IHC+FISH test (33/186 versus 39/186, *P* = ns). There is a decrease of false-negative IHC (3/186 versus 1/186, *P* = ns). This recent 2013 version showed more 2+ IHC (40/186 versus 89/186, *P* = 0.001). For these 2+ IHC, amplification of FISH was only of 14/89 or 15.7%. We have also observed more 3+ IHC (9/186 versus 39/186, *P* = 0.001). One false-positive IHC was described for the 2013 version (0/9 versus 1/26, *P* = ns). Equivocal FISH was also reduced (8/186 versus 2/186, *P* = ns).

Table 2 illustrated that an important part of 1+ IHC of 2007 version became 2+ IHC in the 2013 version (65/118, 55%). Similarly, some 2+ of the 2007 version were reclassified in 3+ IHC in the 2013 version (16/40, 40%).

Table 3 demonstrated the decrease of equivocal FISH and the rise of positive results when we used the 2013 version. When equivocal amplification results were discarded, the Kappa test between the 2 versions was excellent and of 0.96 (95% CI: 0.91–1.0).

The global Kappa test between IHC and FISH was also good for 2007 version of 0.85 (95% CI of 0.68–1.0) and excellent for the 2013 version of 0.96 (95% CI 0.91–1.0).

Table 4 calculated an estimation of the 2 guidelines for 1000 women: for the 2007 version, 180 FISH tests were performed to control 2+ IHC or 300 when the 2+ and 3+ IHC were controlled. In comparison with the 2013 version, 274 FISH tests were performed to control 2+ IHC and 475 when 2+ and 3+ were controlled. The FISH work overflow for the 2013 version was, respectively, +52% for the controls of 2+ IHC and +58% for the controls of 2+ and 3+ IHC.

## 4. Discussion

This retrospective study, based on selected revised IHC and FISH cases, has demonstrated that the 2013 ASCO/CAP guidelines of HER2 evaluation are better than the 2007 recommendations. Indeed, the Kappa agreement test between IHC and FISH was higher for the 2013 version (0.96) than for the 2007 (0.85). No false-positive result was observed with the 2007 system. On the contrary, with the 2013 guidelines, we created some false-positive results. To reduce this rate of false-positive result, some European countries such as Belgium, Germany, or Sweden perform an automatic FISH on all 2+ and 3+ IHC results [9]. The aim of this health politics is based on the fact that false-positive HER2 can lead to treating patients by a potentially toxic and ineffective anti-HER2 therapy. Nevertheless, other authors such as Ross argued that the impact of false-positive HER2 is less important on patients outcome than false-negative results [10]. Also, Rydn et al. calculated that 14.3% of invasive breast cancers were HER2 positive. FISH analysis of 2+ confirmed 12% of amplification and 90% for 3+ [9]. In other words, they found 10% of false-positive HER2 IHC that can be partially explained by the HER2 tumor heterogeneity [11].

Dendukuri et al. discuss the cost effectiveness to test all HER2 2+ and 3+ IHC confirmed by FISH. They found a rate of 282 FISH tests on 1000 women (95% CI: 142–444) [12]. Here, we calculated, for 1000 women, a rate of 300 FISH tests (180 HER 2+ IHC and 120 HER2 3+ IHC) for the 2007 guidelines and 475 FISH tests for the 2013 system that would be retested by FISH (Table 4).

Although our regional HER2 prevalence of 15.1 seems relatively low, recent German multicentric data from 18.081 women has calculated the average HER2-positivity rate of 16.7 ± 3.2% [13]. Also in Sweden the prevalence was 14.3% [9]. These positive HER2 rates were similar to our results.

We also demonstrated that the number of 2+ IHC of the 2013 guidelines was increased twice than that of the 2007, and consequently FISH tests increased similarly (resp., 89/186 for the 2013 version versus 40/186 for the 2007 version). In our experience, low HER2 prevalence involves a tendency of pathologists to reclassify HER2/IHC 1+ to 2+ in order to reduce the number of potential false-negative IHC tests. In other words, the present 2013 recommendation is reassuring for pathologists. Another advantage of the 2013 guidelines is to decrease the number of equivocal FISH amplifications. This problem was recently debated by the French Association of Pathologists that has proposed a HER2 evaluation system, quite similar to the 2013 CAP system for FISH [14]. This GEFPICS's approach is interesting and shows a good correlation with 2013 ASCO/CAP guidelines (excellent Kappa of 0.98, data not shown).

The problem of the HER2/IHC variability is well known. Indeed, HER2 protein is affected by preanalytic steps of the histological procedure such as the tumor cold ischemia, the duration of tissue formaldehyde fixation, the tissue-processing technique, and the paraffin embedding temperature [4, 15]. In a series of 421 cases comparing IHC and FISH with the 2007 guidelines, Vergara-Lluri et al. found a Kappa coefficient of 0.89, similar to our observations. Interestingly, most of their false-negative tests were due to underestimating the IHC 1+, supporting the 2013 guidelines [16].

About FISH, HER2 DNA is more resistant than HER2 protein to tissue alterations caused by preanalytical processes: false-negative results of FISH are rare and false-positive results are associated with the confusing of in situ component in place of the invasive carcinoma. Consequently, Ross et al. proposed FISH method as the primary HER2 screening. This strategy could be justified by an increasing of the accuracy and a more precise use of targeted therapy [17]. Identically, Sauter et al. pointed out that the standardization of IHC in paraffin-embedded tissue is problematic leading to 2% to 8% of false-negative HER2 in IHC 0/1 [18]. These authors argue that primary FISH testing is more cost effective than the evaluation of all IHC-positive patients associated with FISH. The robustness of HER2 FISH was also illustrated by Grimm et al. [19] that described 4% of discordant tests presenting a positive IHC and a negative FISH and explained this discordance more by interpretative errors than by a technical error. The USA pathological practice currently uses FISH as the primary HER2 screening test and IHC as the control test. This methodology does not correspond to the European reality where IHC is the primary HER2 screening test.

The main critic of our study is the selection of patients: 28 cases were discarded, particularly because a technical defect potentially causes false HER2 results, well documented in the literature such as crush artefacts or retraction and small size of tumoral sample [20]. Nevertheless, we did not have the pretention to give epidemiological data and the aim of this study was only to evaluate the impact of the new 2013 recommendation in our European practice. It seems clear that the main change will be an increasing of twice FISH. To reduce this technician time-consuming caused by the FISH work overflow, we used the new rapid FISH technology developed by Dako. This technique was recently evaluated on TMA and approved as similar as the classical FISH method [21]. We point out that, to the best of our knowledge, this study is the first using this new rapid IQFISH Dako system on routine material.

In conclusion, targeted therapy for breast cancer is the new challenge for a personalized medicine and the HER2 FISH or IHC test is the best companion test of reference [22]. Standardization is necessary to treat optimally all candidate patients for a targeted therapy. The new 2013 ASCO/CAP guidelines give us an efficient and robust methodology to realize this aim. Nevertheless, these recommendations could create an increasing of the global cost of the HER2 screening for the laboratories of pathology, mainly caused by the increase of FISH tests, more expensive than IHC and more time-consuming. Finally, further large study could surely need to estimate the true cost effective/benefit ratio of the new ASCO/CAP HER2 guidelines on our European practice.

## Figures and Tables

**Table 1:** Comparison between the 2007 recommendations system and the 2013 updated recommendations.

	FISH							
	2007 guidelines				2013 guidelines			
	E	N	P	Total	E	N	P	Total
IHC								
0+	0	19	0	**19**	0	17	0	**17**
1+	7	108	3	**118**	0	53	1	**54**
2+	1	18	21	**40**	1	74	14	**89**
3+	0	0	9	**9**	1	1	24	**26**
Total	**8**	**145**	**33**	**186**	**2**	**145**	**39**	**186**

The data illustrates the increasing of 2+ IHC (resp., for 2007 and 2013 guidelines of 40 versus 89), 3+ IHC (9 versus 26), and positive HER2 FISH (33 versus 39). There are less equivocal FISH results (8 versus 2) and more false-positive 3+ IHC (0 versus 1).

FISH: fluorescence in situ hybridization, IHC: immunohistochemistry, E: equivocal, N: negative, and P: positive main changes being the increasing of 2+ and 3+ IHC or amplified HER2 FISH.

**Table 2:** Comparison and correspondence between 2007 and 2013 recommendations systems for immunohistochemistry.

		2013 guidelines				
		0+	1+	2+	3+	Total
2007 guidelines	0+	17	2	0	0	**19**
	1+	0	52	65	1	**118**
	2+	0	0	24	16	**40**
	3+	0	0	0	9	**9**
	Total	**17**	**54**	**89**	**26**	**186**

The data shows the changes of 1+ IHC to 2+ IHC (*n* = 65) and the 2+ IHC to 3+ IHC (*n* = 16), respectively, between the 2007 and the 2013 recommendations.

**Table 3:** Comparison and correspondence between 2007 and 2013 recommendations systems for fluorescence in situ hybridization.

		2013 guidelines			
		E	N	P	Total
2007 guidelines	E	1	3	4	**8**
	N	1	145	2	**148**
	P	0	0	33	**33**
	Total	**2**	**148**	**39**	**186**

Kappa test between the 2 ASCO/CAP versions is excellent: 0.96 (95% CI: 0.91–1.0). There are less equivocal results with the 2013 recommendations.

E: equivocal, N: negative, and P: positive.

Note: the decrease of equivocal FISH of the 2013. Two negative cases became positive because the means of HER2 gene were more than 4 copies.

**Table 4:** Example of FISH work overflow calculated for 1000 women.

Indications of the FISH test	2007 guidelines	2007 guidelines	2013 guidelines	2013 guidelines	2013 guidelines	
	2+ IHC	2+ and 3+	2+	2+ and 3+	All cases	
IHC 0+/1+	700	700	525	525	—	(1)
IHC 2+	180	180	274	274	—	(2)
IHC 3+	120	120	201	201	—	(3)
FISH +	36	36	43	236	236	(4)
Number of false-positive IHC 3+/1000 cases	0	0	8	0	—	(5)
Prevalence of HER2	15.6%	15.6%	24.4%	23.6%	23.6%	
Number of IHC performed	1000	1000	1000	1000	0	
Number of FISH tests performed	180	300	274	475	1000	
Net FISH overwork with 2007 as reference (%) for FISH controlling 2+ IHC	0%	+66%	+52%	+163%	+455%	
Net FISH overwork with 2007 as reference (%) for FISH controlling 2+ and 3+ IHC	−40%	0%	−8%	+58%	+233%	

FISH work overflow calculations for 1000 women when we use the 2007 or the 2013 ASCO/CAP recommendations with HER2 FISH as control of 2+ IHC or 2+/3+ IHC (system used in some European countries). The last column illustrated a system of HER2 screening only based on HER2 FISH as used in the USA.

(1) For the 2007 system, 350 cases of 0+ IHC and 350 cases of 1+ IHC are based on the 69.8% of our 0/1+ IHC of our clinical data. Table 1 showed that 54/118 cases (about 50%) of 1+ became 2+ with the 2013 system, about 350 ∗ 0.5 = 175 cases.

(2) Table 1 showed that 16/40 (40%) of the 2+ became 3+ for the 2013 system. For 1000 cases, 180 × 0.4 = 81 cases. For 2013 system, 180 − 81 + 175 = 274.

(3) For the 2013 system, 3+ are the sum of 120 cases of 3+IHC of the 2007 system and 81 cases of the 2+ IHC of the 2007 system that changes in 3+ IHC in the 2013 system. The total of 3+ IHC for the 2013 system is 201 cases.

(4) For the 2007 system, 20% of our 2+ are amplified (20/40) and, for 2013 system, 15.7% of 2+ are amplified (14/89).

In the column of 2013 guidelines of FISH for 2+ and 3+, 240 is calculated for 201 IHC 3+ − 8 false-positive cases (see 5) + 43 cases of 2+ IHC positive FISH.

(5) Table 1: 1/25 or 4% is the rate of false-positive rate for 3+ IHC of the 2013 system. For 1000 cases, there are 201 3+ IHC or 8 false-positive cases (201 × 0.4).
